# Supplementary figures and images for: Microglial senescence contributes to female-biased neuroinflammation in the aging mouse hippocampus: implications for Alzheimer’s disease
Source: J Neuroinflammation. 2023 Aug 16;20:188. doi: 10.1186/s12974-023-02870-2 (PMC10433617; doi:10.1186/s12974-023-02870-2)

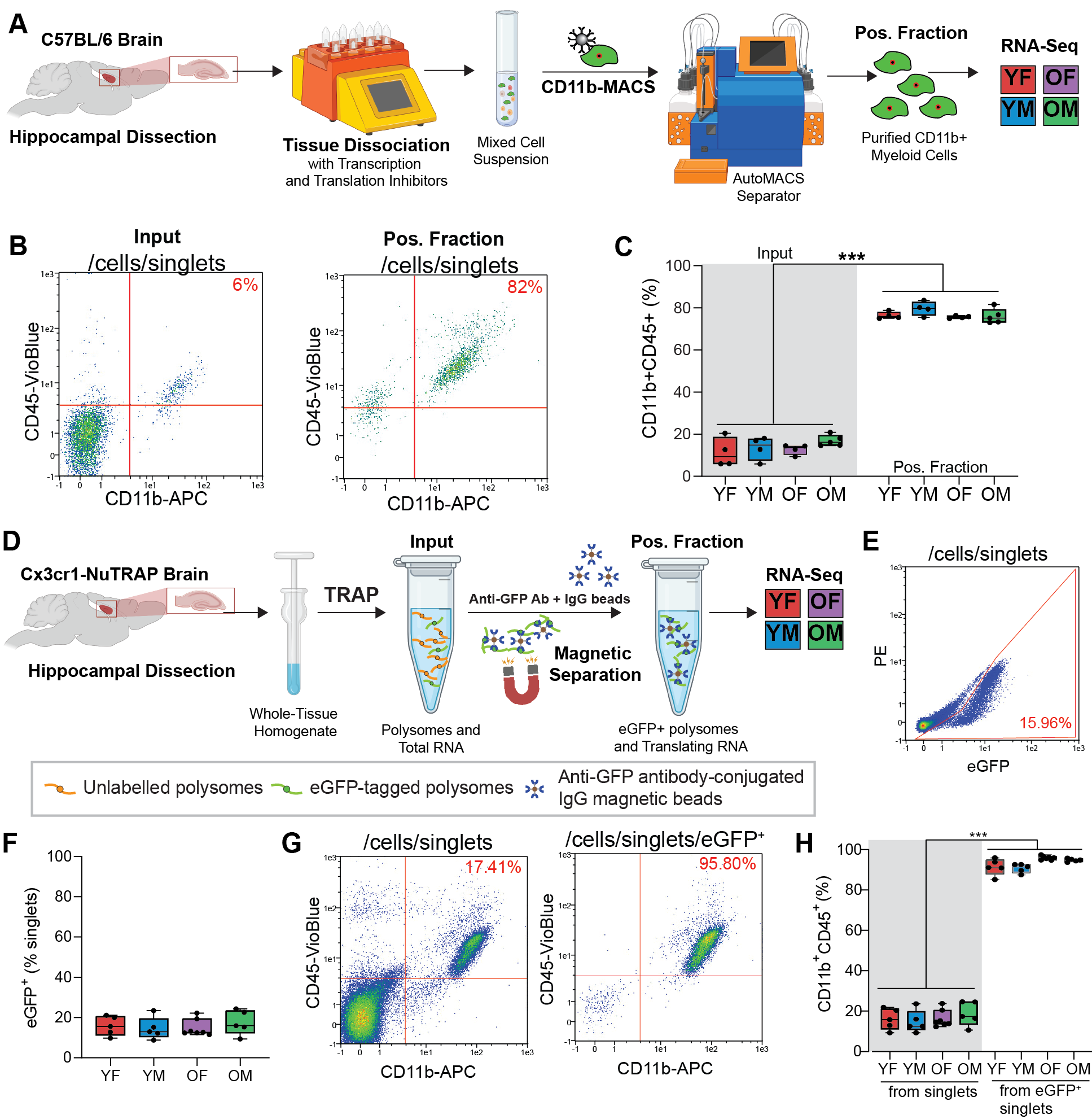

Supplement: Supplementary file 1 — Additional file 1: Figure S1. Isolation of hippocampal microglial transcripts by CD11b-MACS and Cx3cr1-TRAP. A) Isolation of hippocampal microglia by CD11b-MACS. The hippocampus from YF, YM, OF, and OM C57BL6/N mice was dissociated by enzymatic and mechanical dissociation with transcription and translation inhibitors. The single-cell suspension was labeled with CD11b microbeads prior to magnetic separation. The CD11b+ fraction was then analyzed by flow cytometry. B) Representative flow cytometry plots of the CD11b and CD45 immunoreactivity from the CD11b-MACS input and positive (pos.) fractions. C) Quantitation of the percentage of singlets that were CD11b+CD45+ from the CD11b-MACS input and pos. fraction (two-way ANOVA, main effect MACS fraction [Input v. Pos. Fraction], ***p < 0.001). D) Isolation of hippocampal microglial translatome by Cx3cr1-TRAP. Mouse hippocampus was homogenized in TRAP lysis buffer containing translation inhibitors. eGFP-labeled polysomes and associated translating RNA (from Cx3cr1+ cells) were magnetically separated using an eGFP antibody and IgG beads. RNA from the pos. fraction was used to generate stranded RNA-Seq libraries for assessment of the microglial translatome. E–H) Cx3cr1-NuTRAP cortex samples were used to assess the cell specificity of cre-mediated induction of the NuTRAP allele by flow cytometry. E) Representative flow cytometry plot of eGFP+ singlets from Cx3cr1-NuTRAP cortex. F) Quantitation of the percent eGFP+ singlets from young and old Cx3cr1-NuTRAP cortex from both sexes. G) Representative flow cytometry plots of the percent CD11b+CD45+ singlets (left) and eGFP+ singlets (right). H) Quantitation of the percent CD11b+CD45+ singlets (left) and eGFP+ singlets (right). [file 12974_2023_2870_MOESM1_ESM.png]

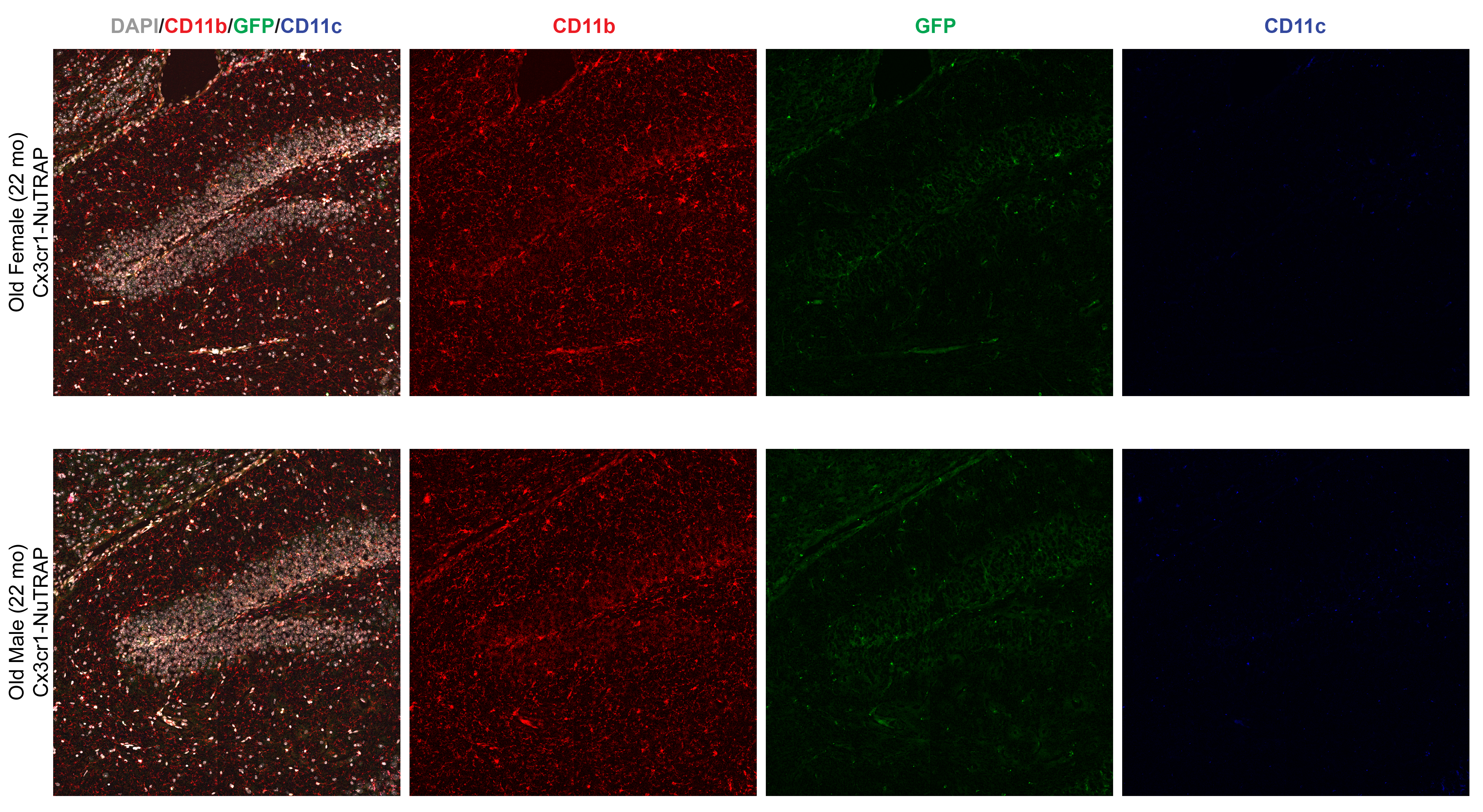

Supplement: Supplementary file 2 — Additional file 2: Figure S2. Separated channels for IHC images displayed in Fig. 5. Cx3cr1-NuTRAP brains were processed for IHC analyses of frozen sections immunostained with antibodies against CD11b (red signal) and CD11c (blue signal). DAPI counterstaining of nuclei is indicated in grey. For the merged images presented in Fig. 5, the eGFP channel was eliminated and the CD11c staining was pseudo-colored to green for better visualization of the colocalization of CD11b and CD11c, and DAPI was colored blue. [file 12974_2023_2870_MOESM2_ESM.png]

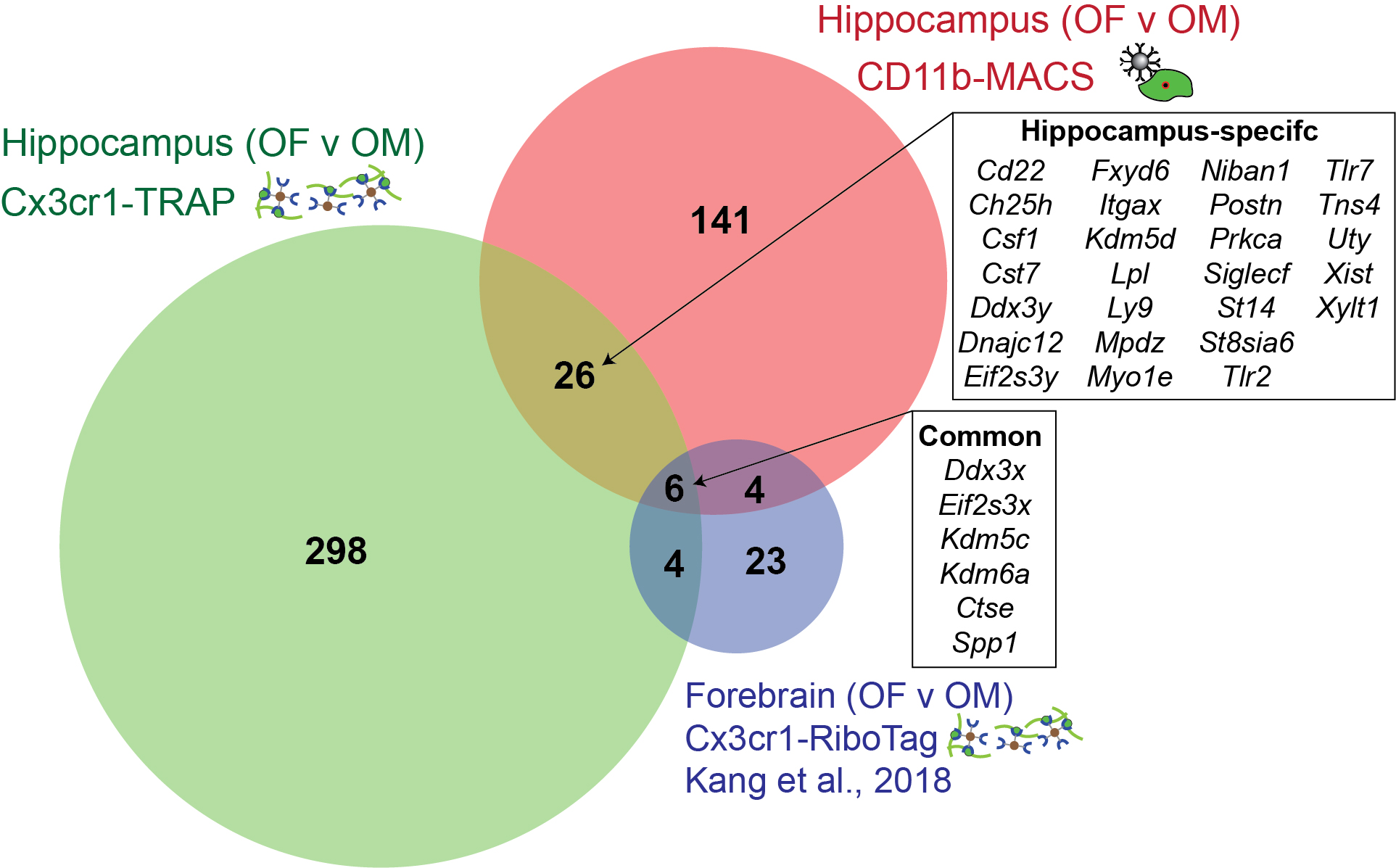

Supplement: Supplementary file 3 — Additional file 3: Figure S3. Comparison of sex effects in aged microglia (22–25 mo) from the present study (hippocampus) and a previously published study (Kang et al., 2018; forebrain) identifies hippocampus-specific sex effects. [file 12974_2023_2870_MOESM3_ESM.jpg]

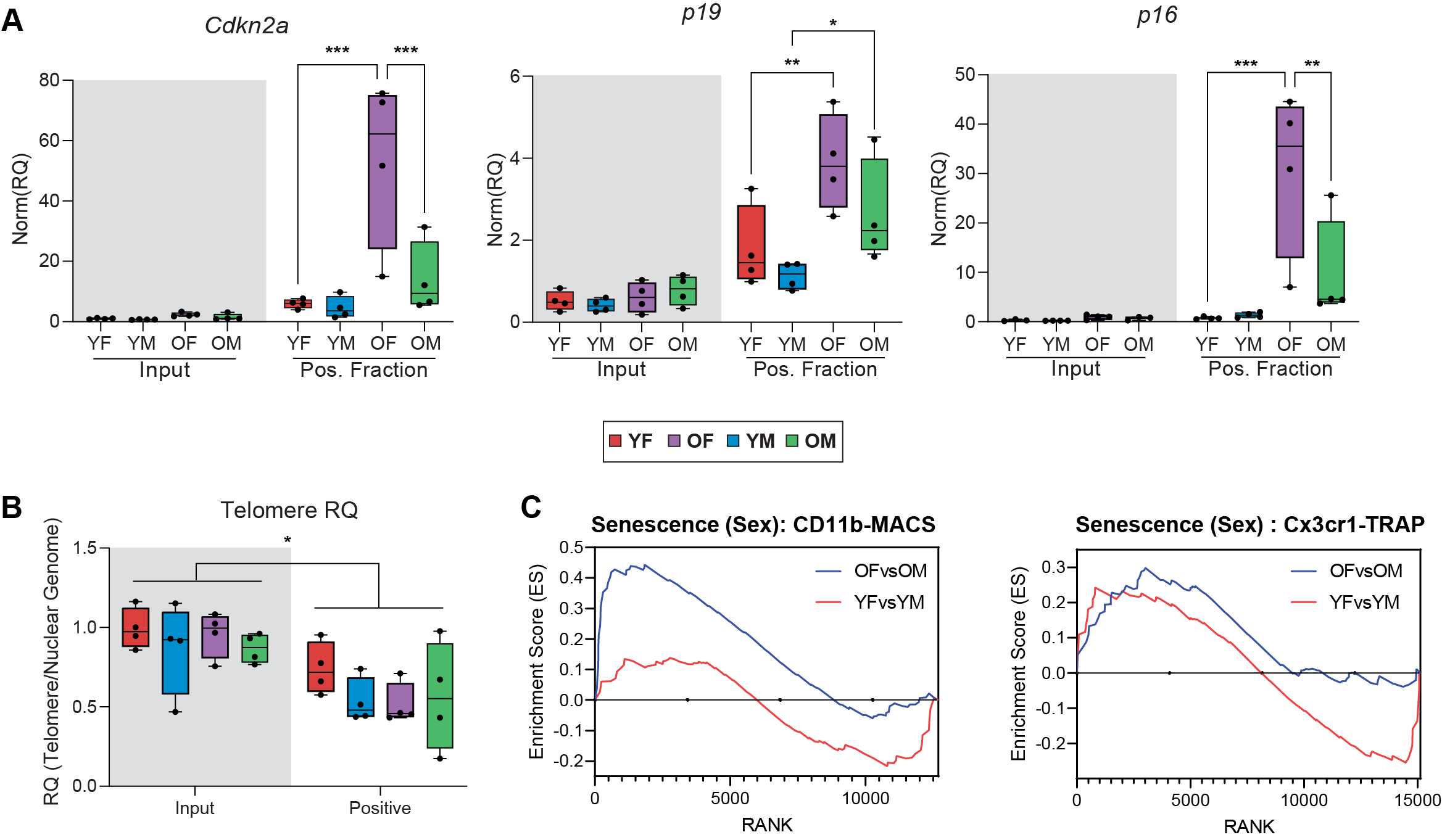

Supplement: Supplementary file 4 — Additional file 4: Figure S4. Senescent marker measurement from the Cx3cr1-NuTRAP brain. A separate cohort of male and female Cx3cr1-NuTRAP mice were aged to 8–13 mo and 23–26 mo (n = 4/sex/age) for TRAP-RT-qPCR and INTACT telomere assays. A) RT-qPCR for Cdkn2a and its transcripts p19 and p16 (two-way ANOVA, Tukey’s post hoc, *p < 0.05, **p < 0.01, ***p < 0.001). B) Relative telomere length as assessed by RT-qPCR (two-way ANOVA, main effect of INTACT fraction (input v. positive), *p < 0.05). Box plots represent median ± IQR. C) GSEA plots showing an enrichment of senescent marker genes in old female microglia compared to old males. [file 12974_2023_2870_MOESM4_ESM.png]
